# Platelet response to influenza vaccination reflects effects of aging

**DOI:** 10.1111/acel.13749

**Published:** 2023-01-19

**Authors:** Anna Konstorum, Subhasis Mohanty, Yujiao Zhao, Anthony Melillo, Brent Vander Wyk, Allison Nelson, Sui Tsang, Tamara P. Blevins, Robert B. Belshe, Daniel G. Chawla, Matthew T. Rondina, Thomas M. Gill, Ruth R. Montgomery, Heather G. Allore, Steven H. Kleinstein, Albert C. Shaw

**Affiliations:** ^1^ Department of Pathology Yale School of Medicine New Haven Connecticut USA; ^2^ Department of Internal Medicine, Section of Infectious Diseases Yale School of Medicine New Haven Connecticut USA; ^3^ Section of Rheumatology, Department of Internal Medicine Yale School of Medicine New Haven Connecticut USA; ^4^ Department of Internal Medicine, Section of Geriatrics and Program on Aging Yale School of Medicine New Haven Connecticut USA; ^5^ Division of Infectious Diseases, Department of Medicine Saint Louis University School of Medicine St. Louis Missouri USA; ^6^ Program in Computational Biology and Bioinformatics Yale University New Haven Connecticut USA; ^7^ Departments of Internal Medicine and Pathology, and the Molecular Medicine Program University of Utah Health Salt Lake City Utah USA; ^8^ Department of Medicine and the GRECC George E. Wahlen VAMC Salt Lake City Utah USA

**Keywords:** age‐specific immunity, flow cytometry, frailty, immunosenescence, platelets, RNASeq, tensor decomposition, vaccination

## Abstract

Platelets are uniquely positioned as mediators of not only hemostasis but also innate immunity. However, how age and geriatric conditions such as frailty influence platelet function during an immune response remains unclear. We assessed the platelet transcriptome at baseline and following influenza vaccination in Younger (age 21–35) and Older (age ≥65) adults (including community‐dwelling individuals who were largely non‐frail and skilled nursing facility (SNF)‐resident adults who nearly all met criteria for frailty). Prior to vaccination, we observed an age‐associated increase in the expression of platelet activation and mitochondrial RNAs and decrease in RNAs encoding proteins mediating translation. Age‐associated differences were also identified in post‐vaccination response trajectories over 28 days. Using tensor decomposition analysis, we found increasing RNA expression of genes in platelet activation pathways in young participants, but decreasing levels in (SNF)‐resident adults. Translation RNA trajectories were inversely correlated with these activation pathways. Enhanced platelet activation was found in community‐dwelling older adults at the protein level, compared to young individuals both prior to and post‐vaccination; whereas SNF residents showed decreased platelet activation compared to community‐dwelling older adults that could reflect the influence of decreased translation RNA expression. Our results reveal alterations in the platelet transcriptome and activation responses that may contribute to age‐associated chronic inflammation and the increased incidence of thrombotic and pro‐inflammatory diseases in older adults.

## Graphical abstract

Platelets mediate innate immune responses as well as thrombosis. We found an age‐associated increase in platelet activation RNAs, further increased in skilled nursing facility (enriched for frailty) vs. community‐dwelling (largely non‐frail) older adults. Tensor decomposition analysis revealed distinct trajectories of platelet activation following high‐dose influenza vaccine. Our results implicate platelets, which are anucleate and lack nuclear transcription, in age‐related chronic inflammation.

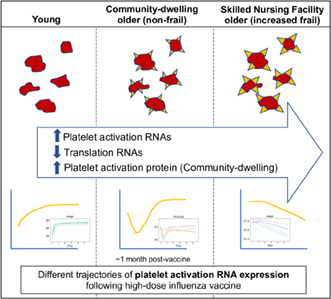

## 1 INTRODUCTION

Substantial evidence indicates that aging is associated with a heightened systemic chronic inflammatory state (Chung et al., 2019; Fulop et al., 2021); the consequences of such dysregulated inflammation include heightened risks for age‐related diseases such as metabolic syndromes, diabetes, cardiovascular disease, and neurodegenerative disease (Furman et al., 2019; Shaw et al., 2013). In addition, chronic inflammation is also associated with loss of muscle mass, altered mobility and other aspects that define the geriatric syndrome of frailty (Ferrucci & Fabbri, 2018; Soysal et al., 2016). The mechanisms underlying the development of age‐related chronic inflammation remain incompletely understood, but are manifested by elevated levels of acute phase reactants, clotting factors, and cytokines. Several interrelated mechanisms underlying this inflammation have been hypothesized, including activation of innate immune pattern recognition receptor (PRR) signaling by age‐related increases in damage‐associated molecular patterns (DAMPs) (Feldman et al., 2015), the senescence‐associated secretion phenotype (SASP), a secretome that includes pro‐inflammatory cytokines induced by cellular damage (Coppé et al., 2010), and altered innate immune PRR function (Molony et al., 2017; Panda et al., 2010; van Duin et al., 2007a; van Duin et al., 2007b).

Platelets are uniquely positioned as mediators of the age‐associated chronic inflammatory milieu, but are principally known as mediators of coagulation and not a typical focus of studies on the biology of aging. These anucleate cell fragments develop in the bone marrow and may reflect systemic inflammation in the circulation. Several studies show that platelet counts decrease with age (Biino et al., 2013; Segal & Moliterno, 2006), and that platelets from older individuals show elevated levels of the pro‐thrombotic platelet factor 4 (PF4) and β‐thromboglobulin. In addition, responsiveness to *in vitro* stimuli promoting platelet aggregation (such as ADP) has also been reported to increase with age (Bastyr et al., 1990; Kasjanovová & Baláz, 1986; Reading & Rosie, 1980; Sie et al., 1981; Zahavi et al., 1980). Notably, platelets secrete cytokines, chemokines, antimicrobial peptides and numerous additional inflammatory mediators; they also express a full complement of innate immune PRRs including Toll‐like Receptors (TLRs), C‐type lectin receptors such as DC‐SIGN and CLEC‐2, and the NLRP3 inflammasome, as well as Fc and complement receptors. Platelet activation mediates interactions with not only endothelium but also cells of the immune system (such as through the platelet‐intrinsic upregulation of CD40‐ligand (CD40L) and p‐selectin (CD62p, the ligand of PSGL‐1)). In addition, platelet interactions with viral as well as bacterial pathogens (such as in the setting of NETosis) have been described. Thus, platelets are uniquely positioned at the nexus of thrombosis and immunity and as such are likely contributors to age‐associated chronic inflammation (Chaipan et al., 2006; Fried et al., 2021; Guo & Rondina, 2019; Hottz et al., 2013; Maouia et al., 2020; Murthy et al., 2017; Suzuki‐Inoue et al., 2006; Thakar et al., 2015).

Though they lack nuclear transcription, platelets contain mRNA, mitochondria and mitochondrial DNA, and machinery for post‐transcriptional regulation of RNA expression. Our previous studies of PBMC transcriptomics to elucidate the effects of age on gene expression signatures of influenza vaccine response revealed modulation of platelet activation pathways, suggesting a role for platelet genes and the presence of platelet‐leukocyte aggregates (Thakar et al., 2015). Globally, an association has been identified between mRNA content and protein levels in platelets, with higher levels of mRNA predictive of detectable identified protein levels (Rowley & Weyrich, 2013). In addition, an association between elevated interferon‐regulated gene and protein expression with platelet activation was identified for patients with systemic lupus erythematosus (SLE) compared to healthy controls (Lood et al., 2010). Additional studies associating platelet gene expression, surface or serum protein levels, and function have been found in thrombocytopenia (Sun et al., 2007), cardiovascular disease (McManus et al., 2013), and inter‐species differences in platelet function (Rowley et al., 2011). Here, we elucidate alterations in the platelet transcriptome and in platelet activation status at the protein level both at baseline and in response to influenza vaccination in young vs. older adults enriched for frail and non‐frail status.

## 2 RESULTS

We enrolled 28 young adults (age 21–35 years), 20 community‐dwelling older adults (age ≥65 years) (Older (Comm)), and 17 older adults (age ≥65 years) (Older (SNF)) who were residents of a skilled nursing facility (SNF) in greater New Haven, Connecticut. As expected, Older (Comm) adults had more comorbid medical conditions and used more medications than young adults, and the Older (SNF) adults showed a further increase in comorbidities and numbers of medications (Table 1). While young adults were assumed to be non‐frail, Older (Comm) and Older (SNF) adults were assessed for frailty, a geriatric syndrome of decreased reserve in response to physiologic stress that is associated with adverse healthcare outcomes, increased disability, and mortality (Fried et al., 2021). The frailty assessment used a previously validated (Kiely et al., 2009) five‐point scale that included measurements of grip strength and gait speed, as well as assessments of unintentional weight loss, decreased physical activity, and exhaustion using validated instruments; individuals meeting criteria for three or more of these criteria are considered frail, 1 or 2 criteria pre‐frail, and zero non‐frail (Fried et al., 2001). The Older (Comm) individuals were non‐frail (16 of 20) except for four individuals who were classified as pre‐frail, while the Older (SNF) adults were frail (15 of 17) except for two individuals who were classified as pre‐frail. These cohorts, therefore, offered the opportunity to assess the biologic consequences of frailty on platelet function. All participants (including the young adults) received the seasonal high‐dose influenza vaccine, which contains four times the dose of vaccine strain hemagglutinin proteins and is approved for use in adults aged 65 or older. We isolated platelet‐rich plasma (PRP) from blood samples of participants obtained prior to vaccination (Day 0) and at Days 2, 7, and 28 post‐vaccine for isolation of RNA and elucidation of the platelet transcriptome via RNA‐seq. Platelet function was further assessed by flow cytometry at Days 0, 2, and 7 post‐vaccine in a subset of participants where flow cytometry was done immediately after PRP isolation.

**TABLE 1:** Cohort characteristics for RNASeq analysis

*N* = 65	Group		
	Young	Older (Comm)	Older (SNF)
	(*N* = 28)	(*N* = 20)	(*N* = 17)
Age			
Mean (SD)	28.46 (5.46)	72.90 (5.47)	83.53 (11.60)
Biological sex			
Female	18 (64.29%)	11 (55.00%)	09 (52.94%)
Male	10 (35.71%)	09 (45.00%)	08 (47.06%)
Race			
Asian	05 (17.86%)	00 (0.00%)	00 (0.00%)
Black or African‐American	03 (10.71%)	00 (0.00%)	00 (0.00%)
White	20 (71.43%)	20 (100.00%)	17 (100.00%)
Hispanic			
No	25 (89.29%)	20 (100.00%)	17 (100.00%)
Yes	03 (10.71%)	00 (0.00%)	00 (0.00%)
Frailty			
Non‐frail	28 (100.00%)	16 (80.00%)	0 (0.00%)
Pre‐frail	00 (0.00%)	4 (20.00%)	2 (11.76%)
Frail	00 (0.00%)	0 (0.00%)	15 (88.24%)
Count of conditions*			
Mean (SD)	0.39 (0.79)	1.95 (1.82)	4.41 (2.60)
Medication			
Daily Aspirin	1 (3.57%)	11 (55.00%)	8 (47.10%)
NSAIDs	11 (39.29%)	4 (20.00%)	1 (5.89%)
Number of prescription medications			
Mean (SD)	0.71 (1.21)	3.40 (2.16)	9.35 (4.44)

*For count of conditions, only conditions occurring in 5% or greater of the sample were included in the count (Congestive heart failure, Coronary Artery Disease, Arrythmias, High blood pressure, Peripheral vascular disease, Stroke or TIA, Chronic pulmonary disease, Asthma, GERD (Reflux disease), Colitis/Irritable Bowel Disease, Diabetes (requiring medication), Renal insufficiency, Thyroid disease, Anemia).

### 2.1 Platelets in older adults are characterized by pre‐vaccination increased platelet activation and decreased translation pathways

**FIGURE 1:**
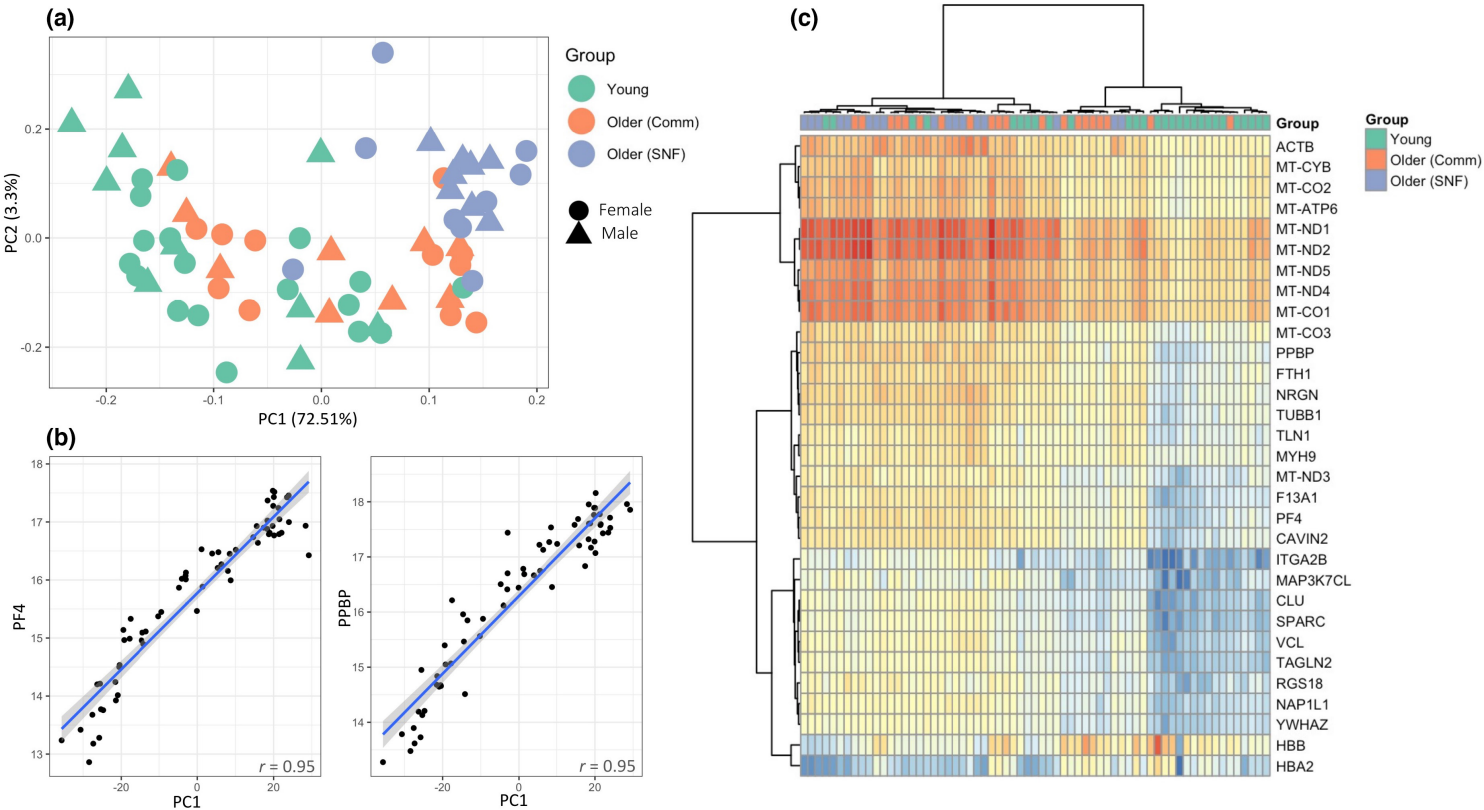
Analysis of RNASeq pre‐vaccination data, (a) PCA using the 500 most variable genes, (b) correlation of PC1 with platelet activation marker expression, (c) expression heatmap of most variable RNAs (RNAs starting with ‘MT‐’ are mitochondrial genes)

We were interested in associations between pre‐vaccination transcriptional profiles and demographic features. The highest proportion of variability in the pre‐vaccination state (~35%) was explained by the group categorization (Young, Older (Comm), or Older (SNF)), with smaller contributions from sequencing run (batch) (~3%) and biological sex (~0.7%) (Figure 1a, Extended Data Figure S1). Overall, pre‐vaccination RNA expression variability was most highly associated with group membership, and there was a continuum of transcriptional states moving from Young to Older (Comm) to Older (SNF) adults.

We performed differential expression (DE) analysis to identify differences in the pre‐vaccination transcriptome among the three groups. This analysis found 1508 RNAs that were significantly different when comparing Young to Older (Comm) adults, and 2209 RNAs that were significantly different when comparing Older (Comm) to Older (SNF) adults (|log2fc| >1.5, adj. *p* value <0.05, Table 2). Platelet activation pathways were significantly enriched in Older (Comm) compared with young adults, as were Rho GTPase effector pathways mediating the activation response (Aslan & McCarty, 2013) and mitochondrial genes (Table 2, Extended Data Figure S2b). Notably, activation pathways in platelets from Older (SNF) adults were also significantly increased compared to Older (Comm) adults. Overall, these results suggest an increase in platelet activation with increasing age and frailty (since nearly all Older (SNF) adults were frail, compared to Older (Comm) adults who were nearly all non‐frail). Indeed, there was a strong correlation of the first principal component (PC1) (which accounted for over 72% of the variance (Figure 1a)) with both age (*r* = 0.63, *p* = 2.12e‐08) and platelet activation (as represented by Platelet Factor‐4 (*PF4*) and Pro‐platelet Basic Protein (*PPBP*, Figure 1b). Notably, *PF4* and *PPBP* were correlated with age (*r* = 0.57, 0.49; *p* = 7.2e‐07, 3.2e‐05, respectively) and frailty (*r* = 0.54, 0.52; *p* = 3.3e‐06; 6.15e‐6, respectively). Interestingly, platelet activation RNA expression was correlated with mitochondrial gene expression across all age groups and several pathways containing mitochondrial genes, including respiratory electron transport and Complex I biogenesis, were enriched in Older (Comm) vs. Young adults (Extended Data Figure S2).

**TABLE 2:** Reactome pathways enriched in pre‐vaccination platelet RNASeq in (a) Young v. Older (Comm) and (b) Older (Comm) v. Older (SNF) individuals

(a) Young v. Older (Comm)		(b) Older (Comm) v. Older (SNF)	
Enriched in Older (Comm) (num. genes = 840)		Enriched in Older (SNF) (num. genes =127)	
Pathway	adj. *p* value	Pathway	adj. *p* value
Hemostasis	2.72E‐24	Hemostasis	1.65E‐05
Platelet activation, signaling and aggregation	2.72E‐24	Platelet activation, signaling and aggregation	2.44E‐05
Platelet degranulation	1.02E‐16	RHO GTPases Activate ROCKs	5.08E‐05
Response to elevated platelet cytosolic Ca2+	3.35E‐16	Platelet degranulation	7.09E‐05
RHO GTPase effectors	2.86E‐13	Response to elevated platelet cytosolic Ca2+	7.91E‐05

Enriched in Young (num. genes = 668)		Enriched in Older (Comm) (num. genes =2082)	
Pathway	adj. *p* value	Pathway	adj. *p* value
Peptide chain elongation	2.19E‐18	Cap‐dependent translation initiation	8.70E‐37
Nonsense‐mediated decay (NMD) independent of the Exon Junction Complex (EJC)	2.19E‐18	GTP hydrolysis and joining of the 60S ribosomal subunit	8.70E‐37
Viral mRNA Translation	2.19E‐18	Formation of a pool of free 40S subunits	8.70E‐37
Selenocysteine synthesis	4.82E‐18	Eukaryotic translation initiation	8.70E‐37
Eukaryotic Translation Elongation	4.82E‐18	L13a‐mediated translational silencing of Ceruloplasmin expression	1.79E‐36

**FIGURE 2:**
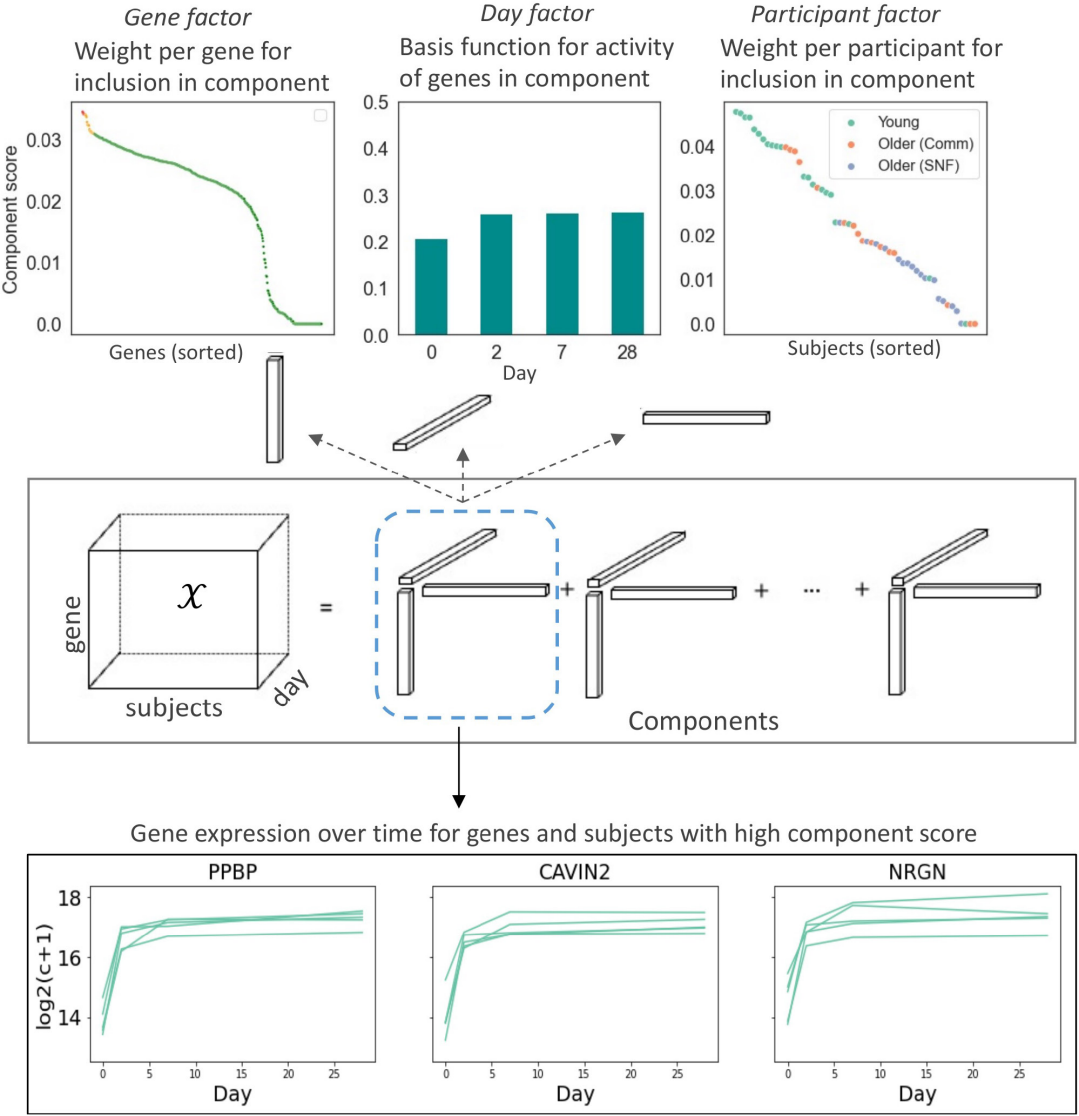
Schematic of non‐negative CP tensor decomposition (NCPD) for platelet vaccine response data. The RNA‐by‐participant‐by‐day tensor is decomposed into components that represent a time‐course pattern of RNA expression for a subset of RNAs across a subset of participants. Such components can be correlated with activation or deactivation of pathways in specific groups

In contrast to the age‐associated increase in platelet activation RNAs, RNAs in pathways related to translation decreased with age and SNF resident status. These pathways were enriched in Young compared to Older (Comm) and Older (Comm) compared to Older (SNF) adults (Table 2, Tables S1–S4). The use of anti‐platelet medications such as non‐steroidal anti‐inflammatory agents (mainly in the young adults) or aspirin (mainly in the older groups) did not appear to substantially influence these findings, as they were preserved on evaluation of the subset of individuals not on any of these medications, despite the loss in statistical power (Tables S5–S6). Additionally, a comparison of male versus female differences did not result in any significantly differentially expressed RNAs when all age groups were considered. Taken together, these findings indicate an age‐associated increase in expression of platelet activation RNAs and mitochondrial genes and a decrease in expression of RNAs encoding translation‐related proteins.

Influenza vaccine antibody response was measured using a standard hemagglutination inhibition assay (HAI). To correct for the inverse correlation between pre‐vaccine HAI titer and fold‐increase post‐vaccine, we employed maximum residual after baseline adjustment (maxRBA), a metric which models HAI titer fold changes as an exponential function of strain‐specific baseline titers and selects the maximum residual across strains (Avey et al., 2020) (Figure S1). maxRBA was used to classify high and low vaccine responders for assessment of platelet transcriptomic differences in vaccine responsiveness. In order to understand how pre‐vaccination transcriptional state is associated with post‐vaccination antibody response, we performed a DE analysis of high‐ vs. low responders in the pre‐vaccination state, and identified 12 upregulated and 7 down‐regulated RNAs in high responders; upregulated RNAs included *THRB, BLZF1, MPIG6B,* and *CCR1* (Table S7). The expression of *THRB*, a nuclear receptor for thyroid hormone, is intriguing in view of the reported role of thyroid hormone on immune response, particularly in the innate immune system (Montesinos & Pellizas, 2019; Wenzek et al., 2022). The association of *MPIG6B* with vaccine response is notable since it acts as a platelet inhibitory receptor that has also been implicated in early megakaryocyte development (Becker et al., 2022; Newland et al., 2007).

Our previous studies found signatures of high and low vaccine response in PBMCs that differed in different groups (Avey et al., 2020; Thakar et al., 2015), and it has been previously shown that sex differences in immune response are also age dependent (Klein & Flanagan, 2016). We therefore assessed what RNAs were DE expressed between males and females and high and low responders in each group, and found that RNAs DE in platelets of males vs. females and high vs. low responders differed substantially for each group (Tables S8–S10, Figure S2). Examples of RNAs expressed at a lower level in males versus females include the *JUN* oncoprotein in young adults – previously reported to be subject to regulation by testosterone (Furman et al., 2014), and the *MPIG6B* RNA encoding an ITIM‐containing platelet inhibitory protein in the Older (SNF) adults (also upregulated in high responders across all groups as discussed above). Hence, response‐ and sex‐associated differences in pre‐vaccination RNA expression were found to differ between the groups.

### 2.2 Response to vaccination shows age‐specific differential response pathways

To identify patterns within the RNA expression changes in platelets post‐influenza vaccination we employed the tensor‐based decomposition approach non‐negative CP decomposition (NCPD). This approach models the data as a non‐negative sum of rank‐one tensors, or components, each one of which corresponds to a temporal expression pattern shared by a subset of participants (Lim & Comon, 2009) (Figure 2). The number of components/temporal patterns was chosen based on measures of decomposition robustness and model error (see Methods). Each RNA and participant has a component score which represents the strength of association with the component time‐course pattern. NCPD thus generates components that can connect distinct patterns of RNA abundance over time into trajectories and associate them with the different participant groups using the ranked weights assigned to participants for each component. The significance of association can be tested using correlation and association analyses of participant and gene ranked weights in each component against clinical and pathway metadata, respectively.

**FIGURE 3:**
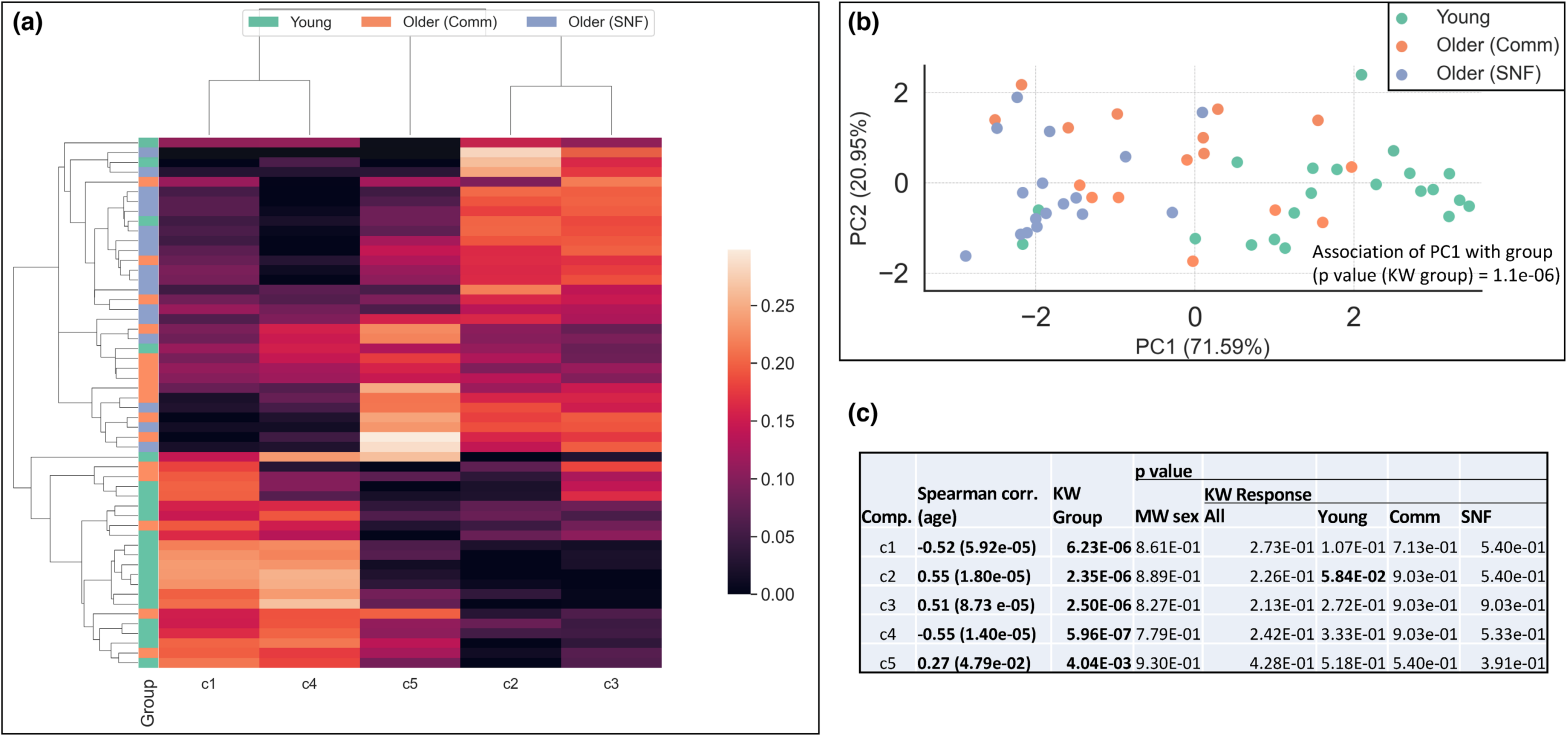
Sample component scores from non‐negative CP tensor decomposition (NCPD) of time‐course platelet transcriptomic response, (a) hierarchical clustering and (b) PCA of sample component scores. (c) Association of component scores with age (Spearman correlation) and group (Kruskall‐Wallis test), biological sex, and vaccine response (Mann‐Whitney *U* test). Bolded values indicate significance of association (*p* < 0.10)

The tensor decomposition identified a model with five components. Participant component scores showed a strong association with the three groups (Figure 3a,b) and were significantly correlated with age and associated with group (Figure 3c). These associations indicate a strong relationship of transcriptomic vaccination response with group and age.

**FIGURE 4:**
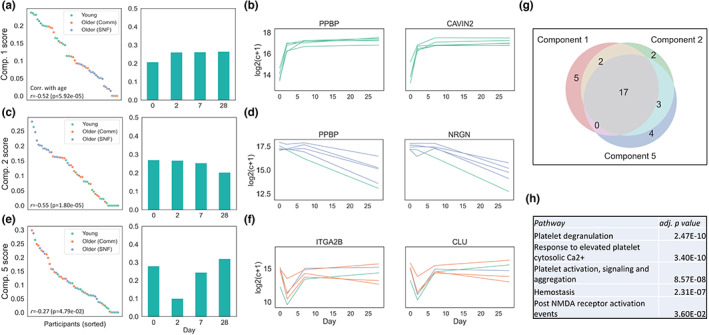
Tensor components related to platelet activation and age group. (a,c,e) Participant and Day scores for Components 1, 2, and 5, respectively, (b,d,f) expression levels for the top 5 scoring participants and top 2 scoring RNAs in each component, (g) Venn diagram of overlapping RNAs in Components 1, 2, and 5, (h) overrepresented Reactome pathways shared by the three components

The top 5% scoring RNAs for Components 1, 2, and 5 were highly overlapping. Pathways involving platelet degranulation, platelet activation, and hemostasis were significantly over‐represented (platelet activation genes *PF4* and *PPBP* were members of this intersecting set). While these components capture similar biology, the temporal patterns and participants associated with each component differed, as reflected in the gene expression patterns of the top scoring participants and genes in each component (Figure 4). Component 1 was associated with young adults, and represents a temporal expression pattern where RNA expression increased post‐vaccination and remained stable. In Component 2, which was associated with Older (SNF) adults, pre‐vaccination RNA levels experienced a sustained drop post‐vaccination and in Component 5, associated with Older (Comm) adults, pre‐vaccination RNA levels experienced a drop followed by a rise back to pre‐vaccination levels. The importance of these RNAs in the distinct temporal response between the groups was further supported by differential expression analysis. All 17 of the overlapping RNAs in Components 1,2, and 5 were significantly differentially expressed between at least one group comparison and post‐vaccination day in a paired sample analysis (Table S12). As an example, if one observes all trajectories across all groups of the gene *PPBP* which was in the intersecting set, the relationship between the group and the distinct temporal trend that is observed in the three components becomes evident. While not all participants within a group have the exact trajectory associated with it, they have the strongest association, as evidenced by the respective component scores (Figure S3). Overall, these results showed that each group displays a distinct temporal pattern of RNAs associated with platelet activation.

RNAs reflecting expression of mitochondrial genes *MT‐1* and *MT‐2* were among the top scoring in Components 1 and 2 (Table S11), indicating that the correlation between mitochondrial gene and platelet activation RNA expression in the pre‐vaccination state may extend post‐vaccination. Indeed, the correlation between platelet activation and mitochondrial gene activity was still strongly present in the time‐course (Figure S4). Top‐scoring RNAs In Components 3 and 4 were also highly overlapping, with pathways involving RNA translation significantly over‐represented. We observed that expression of these RNAs tended to have higher pre‐vaccination levels which then decreased in young adults, while pre‐vaccination levels were lower and increased in Older (SNF) adults, with intermediate dynamics in Older (Comm) adults (Extended Data Figure S3). These RNAs were also found to be significantly differentially expressed in a paired sample analysis (Table S12). Therefore, the negative correlation between platelet activation and translation gene RNA levels observed at pre‐vaccination was maintained following vaccination.

### 2.3 Effects of age and SNF residence on platelet activation at the protein level and comparison with expression of activation marker RNAS following vaccination

**FIGURE 5:**
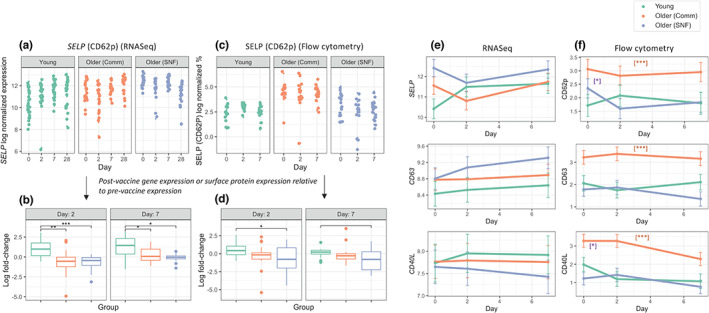
Evaluation of protein and RNA expression of the platelet activation markers p‐selectin (CD62p, encoded by the SELP gene), CD63, and CD40L. (a,c) Scatter plots depicting log‐normalized gene expression for SELP and percent cells positive for SELP (CD62p) as measured by flow cytometry, respectively; (b,d) Log2 fold change at day 2 and 7 post‐vaccine relative to prevaccination levels of SELP gene expression (a) or SELP (CD62p) surface expression (c) in Young (RNASeq, *n* = 28; flow cytometry *n* = 14), Older (Comm) (RNASeq, *n* = 20; flow cytometry, *n* = 17) and Older (SNF) (RNASeq *n* = 17, flow cytometry n = 17) adults; (e,f) Generalized linear mixed effect models for log normalized expression (RNASeq) and percent cells positive (flow cytometry) of the three markers in (e) RNASeq and (f) flow cytometry at prevaccination, and days 2 and 7 post‐vaccination. Significance for (b), (d): ***, *p* < 0.001; **, *p* < 0.01; *, *p* < 0.05; open bracket, *p* < 0.10; significance for (f): orange asterisks indicate the Older (Comm) group was significantly different from Young and Older (SNF) at all days and all time points at adj. *p* value at least <0.01; purple asterisks indicate that Young and Older (SNF) were significantly different at day 0 at adj. *p* value at least <0.05

We assessed the state of platelet activation at the protein level by determining the surface expression of p‐selectin (CD62p, encoded by the *SELP* gene), CD40 Ligand (CD40L) and CD63 by flow cytometry in Young, Older (Comm) and Older (SNF) groups. All three proteins are found in α‐granules (CD62p and CD40L) or dense granules (CD63) within platelets and are transported and expressed on the platelet surface upon activation (André, 2004). We compared these results to the expression of RNAs encoding these proteins to evaluate the relationship between transcription and protein levels of these activation markers (Figure 5).

The levels of surface expression of all three proteins were significantly increased in platelets from Older (Comm), compared to young adults at day 0, 2, and 7, suggesting an age‐associated increase in platelet activation. We expected that the Older (SNF) group might show similar or even increased expression of these activation markers. However, surface expression in Older (SNF) adults was significantly decreased compared to Older (Comm), with Older (SNF) activation marker surface expression comparable to that of platelets from young adults (with the exception of Day 0 CD62p expression in Older (SNF) adults, which was significantly higher compared to platelets from young adults) (Figure 5c,f). The levels of these markers in Older (Comm) adults with or without use of anti‐platelet medication were similarly increased, indicating that use of anti‐platelet medication was not driving this difference.

We found that expression of *SELP* RNA at day 2 and day 7 post‐vaccine, normalized to day 0, followed the pattern of platelet activation RNA levels identified by tensor decomposition analysis (Figure 4), with highest fold‐change in young adults, followed by Older (Comm) and Older (SNF) adults (Figure 5b). Notably, the surface expression of CD62p/SELP protein at these post‐vaccine time points relative to day 0 closely resembled the RNA expression pattern (Figure 5d). We did not observe a similar parallel relationship between normalized RNA expression and protein expression for CD40L and CD63 (Figure 5e,f). These findings suggest a potential link between RNA and protein expression of *SELP* and CD62p, respectively, despite the presence of pre‐formed CD62p within α‐granules. Taken together, these findings demonstrate an age‐associated increase in platelet activation in Older (Comm), compared to young adults, that is attenuated in platelets from Older (SNF) adults.

### 2.4 Young, but not older adults exhibit dynamics of RNA abundance that are associated with antibody response to influenza vaccination

**FIGURE 6:**
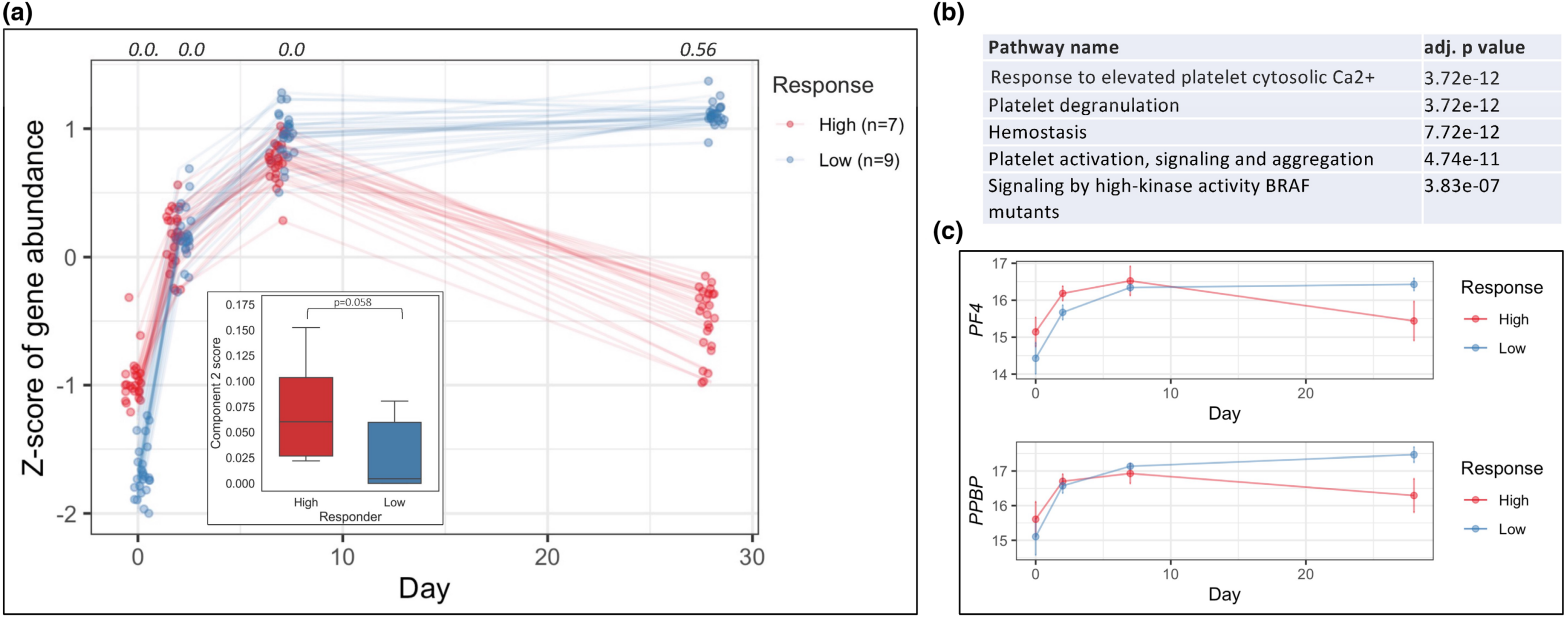
Young adult vaccine high responders show different expression trajectories compared to low responders. (a) Trajectories for high‐ and low responders of top 5% scoring Component 2 RNAs in Young adults. The fraction of RNAs that are significantly differentially expressed from this set listed on top. Inset: Component 2 score for Young high‐ vs. low responders. (b) Over‐represented Reactome pathways for top Component 2 genes, (c) Mean trajectories for Young high‐ and low‐responders for genes *PF4* and *PPBP*

When evaluating component associations against participant characteristics, an association between Component 2 scores and vaccine antibody response in only young participants was observed (*p* = 0.058), we did not find an association between platelet RNA levels and vaccine antibody response in the older adults groups (Figure 2c; Figure 6a, inset). Among young high responders (*n* = 7), Component 2 RNAs decreased in expression on days 7‐28, whereas in low responders (*n* = 9) the expression levels remained stable (Figure 6a). In order to assess the significance of this observed difference, we performed a DE analysis on the set of top 5% scoring RNAs from Component 2 between young adult high‐ and low responders at each day post‐vaccination, and found that more than 50% of the RNAs were significantly DE on day 28, while there were no significant differences at the other timepoints (Figure 6a). These genes include *PF4* and *PPBP* and are enriched in pathways associated with platelet response (Figure 6b,c). To observe whether this phenomenon extended beyond the top‐scoring Component 2 RNA‐set, we performed the DE analysis for all RNAs across the time‐course in young adult high‐ vs. low responders, and found that the greatest number of differentially expressed RNAs were more highly expressed in low responders at day 28. The set of RNAs highly expressed in low responders at day 28 encode for proteins involved in pathways that include Rho GTPase effectors and platelet activation (Extended Data Figure S1,S4). Thus, Component 2 elucidated a difference in time‐course patterns of platelet activation RNAs between high‐ and low young adult responders in the day 7‐28 post‐vaccination period.

## 3 DISCUSSION

We carried out transcriptomic analyses of human platelets from Young, older community‐dwelling (Older (Comm)) and older SNF resident (Older (SNF)) adults in the context of seasonal high‐dose influenza vaccination. Using an operational definition of the geriatric syndrome of frailty allowed us to further compare the Older (Comm) group, which was comprised almost entirely of non‐frail individuals, to the almost exclusively frail Older (SNF) group. Additional covariates, such as BMI and race/ethnicity, were not investigated since the characteristics of enrolled participants (namely the limitations of self‐reported BMI and substantial racial/ethnic diversity found only in the young group) limited the ability to draw conclusions from statistical analyses; these warrant further investigation both individually and in concert with age effects (Barrachina et al., 2019; Edelstein et al., 2013).

Prior to vaccination, we found a marked increase in RNA expression signatures of pathways including platelet signaling, degranulation, and hemostasis in Older (Comm), compared to young adults and additionally in Older (SNF) residents compared to Older (Comm) adults. These findings reveal that the transcriptome of platelets from older adults reflects a general activated, pro‐thrombotic state compared to young adults. Previous analyses of the platelet transcriptome in healthy young and older, community‐dwelling non‐frail adults reported evidence for an age‐associated increase in Granzyme A expression at the mRNA and protein levels, leading to increased leukocyte signaling and cytokine generation (Campbell et al., 2018). Our transcriptomic findings suggest an age‐associated heightened basal activation state in platelets. In addition, the predominance of frailty in the Older (SNF) group and non‐frailty in the Older (Comm) identifies a potential relationship between frailty and increased levels of RNAs associated with platelet activation. We also observed enrichment of pathways associated with mitochondrial genes Older (Comm) vs. young adults, and a correlation between mitochondrial gene RNAs and platelet activation RNA expression across all participants. Notably, because mammalian platelets are anucleate, the mitochondrial genome represents the only endogenous DNA basally present in platelets. Such mitochondrial DNA encodes 13 proteins mediating oxidative phosphorylation, as well as a group of tRNAs and ribosomal RNAs (Taylor & Turnbull, 2005). Previous studies have revealed increased mitochondrial mass and oxygen consumption associated with TNF‐dependent inflammation in aged murine platelets (Davizon‐Castillo et al., 2019); our findings point to a role for mitochondrial dysfunction in aged human platelets as well. We note that the correlation between mitochondrial RNAs and RNAs encoding platelet activation genes, the latter of which are not endogenously generated via transcription in platelets, suggests that the high variability that is observed in both RNA sets results from not only new transcription (for mitochondrial genes) but also post‐transcriptional mechanisms such as differences in RNA stability. Additionally, our analyses of pre‐vaccination platelet RNA expression also revealed a decrease in expression of RNAs related to translation in Older (Comm), compared to young adults, as well as in Older (SNF) compared to Older (Comm) adults. Overall, we observed an increase in platelet activation status at the transcriptomic level from Young to Older (Comm) and Older (Comm) to Older (SNF) adults that was also associated with increased mitochondrial gene expression and decreased levels of RNAs associated with translation. It is attractive to speculate that this combination of increased platelet activation and decreased translation in Older (SNF) vs. Older (Comm), and all older compared to young adults may result in differential functional effects on platelet activation in older adults with or without frailty.

To analyze temporal patterns, we leveraged the CANDECOMP/POLYADIC (CP) decomposition, a tensor decomposition framework that has seen increased application for complex analytics in bioinformatics, including characterizing tissue‐specific gene expression phenotypes (Hore et al., 2016), *Mycobacterium tuberculosis* subtyping (Ozcaglar et al., 2011), and systems serology profiling (Tan et al., 2021). To extract multi‐index patterns that are individually interpretable, we performed non‐negative CP (NCPD), which models the data as a non‐negative sum of rank‐one tensors, termed components (Lim & Comon, 2009). The non‐negativity constraint ensures that the model can be analyzed either as a whole and/or on a component‐by‐component basis since cancellation by negative values will not occur. This is analogous to the strengths exhibited by non‐negative matrix factorization (NMF) (Lee & Seung, 1999), which has been used in a variety of biological applications, including sample clustering and biological module detection (Brunet et al., 2004; Devarajan, 2008). While other decomposition methods for tensor data exist (Tucker, HGSOC) (Kolda & Bader, 2009), NCPD has the advantage that it is both non‐orthogonal, which can allow derivation of patterns with overlapping collinear genes, and generates components for straightforward downstream analysis and association with demographic and clinical attributes.

In contrast to conventional differential gene expression analysis, NCPD is particularly well suited to delineate trajectories of RNA expression over time, in this case, following influenza vaccination. Using a novel application of NCPD, we identified three components corresponding to RNAs that mediate platelet activation, but which showed distinct trajectories among the groups after influenza vaccination – with increased and sustained platelet activation beginning at day 2 in young adults, decreased activation at day 2 followed by an increase in Older (Comm) adults, and a progressive decrease post‐vaccination in Older (SNF) adults (Figure 4). Genes associated with these components also included mitochondrial genes, showing that the association between platelet activation and mitochondrial RNAs identified at pre‐vaccination was maintained following vaccination across groups. We also identified components containing RNAs mediating translation, which demonstrated distinct expression patterns in young adult versus older groups (Extended Data Figure S3). Taken together, these results showed that platelet activation pathways follow distinct temporal trajectories in the different groups following vaccination and are associated with broader changes in the transcriptome.

We further studied the effects of age, SNF vs. Comm status, and influenza vaccination on proteins associated with platelet activation by assessing the basal expression of surface p‐selectin (CD62p, encoded by the *SELP* gene), CD40L, and CD63, prior to and following vaccination using flow cytometry. We found that the change in CD62p/SELP protein expression at day 2 and 7 post‐vaccine, relative to day 0, closely resembled that observed at the same timepoints relative to day 0 for *SELP* RNA expression in Young, Older (Comm), and Older (SNF) adults (Figure 5b,5d). At both time points post‐vaccine, the lowest levels of normalized CD62p surface protein and RNA expression were found in Older (SNF) adults, similar to the generalized decrease in platelet activation RNAs following vaccination seen in this group by NCPD (Figure 4). The parallel trajectories of CD62p/SELP surface expression and *SELP* RNA expression suggest a link between transcription by the platelet parent cell, the megakaryocyte, in the upregulation of surface CD62p protein (Davizon‐Castillo et al., 2020). On the other hand, other mechanisms such as differences in RNA stability may also influence the differential abundance of specific transcripts, perhaps in part explaining why normalized protein expression of CD40L and CD63 at days 2 and 7 did not parallel expression of their corresponding RNAs. The reasons for the different behavior of CD62p/SELP vs. CD40L and CD63 are unclear; however, the fact that CD62p, CD40L, and CD63 surface expression upon platelet activation results from the translocation of preformed protein from alpha granules (CD62p and CD40L) or dense granules and lysosomes (CD63), combined with the cleavage or secretion of platelet‐derived CD40L and CD62p add complexity to the regulation of expression of these platelet activation markers (Cognasse et al., 2015; Henn et al., 2001; Kostelijk et al., 1996).

In analyzing the absolute levels of CD62p, CD40L and CD63 surface expression pre‐ and post‐vaccine, we found that all three activation markers were markedly elevated in platelets from Older (Comm), compared to young adults at baseline and at day 2 and 7 post‐vaccine – consistent with an age‐associated increase in platelet activation (Figure 5). Interestingly, platelets from the Older (SNF) group showed surface expression levels of these markers that were significantly lower than the Older (Comm) individuals and were in fact comparable to the young group (except for a day 0 elevation in CD62p expression that was intermediate between Young and Older (Comm) groups). The basis for this finding remains unclear, and contrasts with the platelet activation RNA expression pattern found in the Older (SNF) group that was markedly increased compared to not only the young group but also the Older (Comm) group. One possibility is that the substantial decrease in RNAs mediating translation found in the older groups – which was lowest in the Older (SNF) adults – suppressed expression of these activation markers. In this regard, previous studies of the effects of age and frailty on platelet CD62p basal surface expression showed both increased CD62p expression in frail vs. non‐frail adults (Arauna et al., 2020) as well as trends that were similar to our findings (Hernández et al., 2019). In addition, an age‐associated increase in platelet oxidative stress reported in participants aged 40–79 appeared to be reversed in individuals aged 80 and above (Jain et al., 2019). In sum, we found enhanced levels of RNAs associated with activation and mitochondrial gene expression, combined with decreased expression of RNAs mediating translation in older adults, including increased platelet activation RNAs in Older (SNF) adults enriched for frailty compared to largely non‐frail Older (Comm) individuals. We found evidence for enhanced platelet activation at the protein level in Older (Comm) adults that was attenuated in Older (SNF) adults, perhaps reflecting decreased expression of translation RNAs. Our findings suggest alterations in the platelet transcriptome, proteome and activation responses in both non‐frail and frail older adults. These changes would be predicted to generally promote a thrombo‐inflammatory milieu and may contribute to the increased risk of thrombotic and inflammatory diseases in non‐frail and frail older adults.

The basis for age‐associated alterations in the platelet transcriptome is particularly intriguing since platelets are anucleate and, except for the mitochondrial genome, lack transcriptional activity. As discussed, the possibility that these age‐related differences are platelet‐intrinsic could reflect differences in RNAs expressed in precursor megakaryocytes that generate platelets. Consistent with this hypothesis, other groups have reported that induced changes in platelet RNA expression can be explained, in part, by increased investment of RNA into developing platelets by megakaryocytes (Campbell et al., 2019; Middleton et al., 2019). Non‐cell intrinsic mechanisms such as regulation of post‐transcriptional effects on RNA stability by inflammatory stimuli could also play a role (Akira & Maeda, 2021); in this context, our finding that influenza vaccine response in young (but not older) adults was associated with decreased expression of RNAs enriched for platelet activation genes at day 28 post‐vaccine would be consistent with this idea (Figure 6), since typical human platelet lifespan is approximately 8–10 days (Harker et al., 2000; Leeksma & Cohen, 1955). Further studies to address this question would provide insights that could be highly relevant to ameliorating not only the substantial burden of cardiovascular and other thrombotic diseases, but also the increased thrombotic complications associated with conditions such as COVID‐19 in older adults.

## 4 METHODS

### 4.1 Human participants

This study was conducted in accordance with guidelines approved by the Human Investigations Committees of Yale School of Medicine with written informed consent approved annually. We enrolled 28 young adults (age 21–35 years), 20 community‐dwelling older adults (age ≥65 years) (Older (Comm)), and 17 older adults (age ≥65 years) (Older (SNF)) who were residents of a skilled nursing facility (SNF) in greater New Haven, Connecticut (Table 1). Study participants had no acute illness and took no antibiotics within one month of enrollment. Demographic characteristics of participants were collected at enrollment (Table 1). Self‐reported information included demographic data, height, weight, medications, and comorbid conditions; immunocompromised individuals were excluded as described previously (Mohanty et al., 2015). Participants were enrolled during the 2018–2019 influenza vaccine season. All participants received the high‐dose trivalent influenza vaccine used in that season (Fluzone High‐Dose) containing hemagglutinin (HA) proteins from A/Michigan/45/2015 X‐275 (H1N1), A/Singapore/INFIMH‐16‐0019/2016 IVR‐186 (H3N2), and B/Maryland/15/2016 BX‐69A (a B/Colorado/6/2017‐like virus, B Victoria lineage) at a dose of 60 μg for each HA. Blood samples were collected prior to vaccination (day 0) and follow‐up days 2, 7, and 28. Antibody response to vaccination was assessed using serum samples obtained at day 0 and 28 using a standard hemagglutination inhibition assay (Frey et al., 2010).

### 4.2 Platelet preparation

About 8 ml of blood was collected in acid citrate dextrose (ACD) tubes (Cat. Number 364606, BD Biosciences) for platelet isolation. To avoid shear forces impacting platelet activation, the ACD tube was not drawn first during blood collection. Samples were kept at room temperature and centrifuged at 240 × *g* using a bench top centrifuge (Thermo Fisher Scientific) for 20 min without brake. The straw‐colored platelet rich plasma (PRP) was carefully transferred to a 15 ml conical tube for RNA extraction and flow cytometry.

### 4.3 Flow cytometry

An antibody cocktail for platelet flow cytometry analyses included CD61 FITC (Cat. Number 336404), CD40L PE (Cat. Number 310806, Bio legend), CD14 PE‐CF594 (Cat. Number 562335, BD Biosciences), CD63 PercpCy5.5 (Cat. Number 353022, Bio legend), CD41 Alexa Fluor 700 (Cat. Number 303728, Bio legend), CD62p PECy7 (Cat. Number 304922, Bio legend), CD45 APCCy7 (Cat. Number 3368516, Bio legend) and CD66b Pacific Blue (Cat. Number 561649, BD Biosciences). About 100 μl of PRP was mixed with 100 μl of antibody cocktail. After incubation for 20 minutes at room temperature, samples were washed with 1x FACS buffer (1x PBS containing 2% FBS) followed by a paraformaldehyde (PFA) fixation step involving BD Cytofix buffer for 10 min at room temperature. Samples are washed with 1X FACS buffer again to remove the PFA and finally re‐suspended in 1 × FACS buffer for flow cytometry analysis using either Fortessa instrument (Becton Dickinson) or CytoFlex LX instrument (Beckman Coulter) fitted with an automated sampler accommodating 96‐well plates. FCS files generated by the BD FACS DeVa software (Bd Bio Sciences) or CytExpert software (Beckman Coulter) were analyzed using FlowJo software V10. (FlowJo, LLC). Particles recorded in log scale forward and side scatter (FCS and SSC) were distinguished as anucleate platelets by the surface expression of CD41+ and CD61+. The activation status of platelets was further estimated as percentage of CD41+ CD40L+, CD41+CD62P+ and CD41+CD63+ particles. Samples with excessive aggregation (indistinguishable CD41+CD61+ population) during sample preparation and subsequent staining were excluded from flow cytometry analysis using FlowJo software V10. (FlowJo, LLC).

### 4.4 maxRBA calculation

Changes in serology were quantified and vaccine response groups defined using the maximum residual after baseline adjustment (maxRBA) method (Avey et al., 2020). Briefly, this approach fits an exponential model to predict titer fold change using baseline titer values for each vaccine strain separately. A participantt's maxRBA score is the maximum residual across all measured vaccine strains for that individual. MaxRBA scores were discretized using quantile cutoffs: those equal to or below the bottom 40th percentile were classified as low responders and those above the top 40th percentile were classified as high responders. We applied this method to generate participant labels separately in the Young and Older(Comm) + Older (SNF) groups.

### 4.5 RNA preparation

About 500 μl PRP was added to 700 μl of QIAsol lysis reagent (cat. 217004, Qiagen) and mixed by pipetting at least 10 times to ensure proper lysis. Lysed PRP samples were immediately frozen at −80°C until further extraction. RNA samples were prepared using the miRNeasy kit (cat. 217004, Qiagen) following the manufacturer's instructions. Briefly, PRP lysed in QIAzol reagent was incubated for 5 min at room temperature. To each sample 140 μl of chloroform was added and mixed vigorously and left at room temperature for about 5 min. Subsequently, samples were centrifuged at 4°C at 12,000 × *g* for 15 min. The upper aqueous phase containing RNA was carefully transferred to a 2 ml collection tube (cat. 990381, Qiagen) without touching the interphase and placed in a QIAcube instrument for extraction.RNA extraction was carried out using the recommended protocol (FIW‐50‐001‐J_FW_MB and PLC program version FIW‐50‐002‐G_PLC_MB) available from the QIAcube web portal. RNA samples with RNA Integrity Number (RIN) values above 7.0 were used for RNA expression analysis.

RNA‐seq libraries were prepared using the Takara Bio SMARTer Stranded Total RNA‐Seq‐kit ‐ Pico Input Mammalian per the manufacturer's instructions. Libraries were sequenced on an Illumina NovaSeq 6000, S4 flowcell, 2 × 100 paired‐end, following the manufacturer's protocols. Low quality reads and reads with length <50 bp were removed using Trimmomatic v 0.36. Base‐quality was then assessed with FastQC v 0.11.7. FASTQ files were aligned using STAR V 2.7.3, against human reference GRCh38p12. Gene counts were determined using HtSeq‐count and gencode.v30.chr_patch_hapl_scaff.annotation.gtf. Data for each sample and participant are available via ImmPort (https://www.immport.org) under accession number SDY1393. Raw and count data has been submitted to the Gene Expression Omnibus Database (https://www.ncbi.nlm.nih.gov/geo/) with accession number GSE178158.

### 4.6 RNASeq processing

The data was pre‐processed to only include protein‐coding genes and exclude genes on the X and Y chromosome. Following, genes were filtered for low expression by (1) removing genes with non‐zero counts in less than three samples (which corresponds to approximately 1% of total samples) and (2) filtering out the bottom 10% expressing genes of the remainder. Approximately 16,800 genes were kept for analysis following pre‐processing.

### 4.7 DESeq Model‐based analyses

DESeq2 (Love et al., 2014) was used to normalize gene counts prior to performing PCA and hierarchical clustering on the baseline data, as well as for differential expression analysis. For DESeq2 analysis of pre‐vaccination data, a Principal Variance Component Analysis (PVCA) was performed on all the groups, as well as each age group separately as it was expected that there would exist heterogeneity in the covariate contribution to the variation, to assess covariates to include in the model (Boedigheimer et al., 2008; Bushel, 2022) (Extended Data Figure S1). For the anti‐platelet medication covariate, NSAID use was used for young adults, and daily aspirin or prescription anti‐platelet medication (‘dAspirinplus’) for the Older (Comm) and Older (SNF) adults, since these were the predominantly used anti‐platelet medications used in the respective groups (Table 1). Covariates that were present in more than 2 participants were included in the PVCA and models. Batch refers to sequencing run, and cohort for the young adults refers to the location of sample collection for the participants.

Based on the results of the PVCA, the model designs were set as follows:

All participants: ~ Group + Biological.sex + Batch,

Young: ~Biological.sex + Cohort + NSAIDs + Batch,

Older (Comm): ~Biological.sex + Frailty + dAspirinplus + Batch,

Older (SNF): ~Biological.sex + dAspirinplus + Batch,

Genes considered as DE had to have a (i) |logFC|≥1.5, (ii) Benjamini‐Hochberg adj. *p* value <0.05 (unless otherwise noted), (iii) ≥25% of samples compared expressing the gene. The latter requirement was necessary in order to exclude genes that were only expressed in a small number of individuals, generally at low counts, which led to very high logFC differences. The functional enrichment analysis on genes considered DE between selected groups was performed on the Reactome database (Jassal et al., 2020) using g:Profiler (version e104_eg51_p15_3922dba) and Benjamini‐Hochberg false discovery rate correction (Raudvere et al., 2019). To assess whether the higher frequency of daily Aspirin use in Older adults may have impacted the group‐specific differences, we ran the model design All participants: ~Group + Biological.Sex + Batch on participants that did not use any anti‐platelet medication.

DE analysis models for the time‐course were built using a multi‐level, repeated measures design. Comparisons were made at each day across age groups, and for young individuals, across high‐ and low responders.

### 4.8 Tensor decomposition

Genes for CP decomposition were filtered more stringently than for DESeq2 analysis since the former does not provide an additional mechanism to address low‐expressed genes. Genes were filtered to remove genes with counts ≤100 in ≥30 samples (which corresponds to approximately 12% of total samples), and the bottom 10% expressing genes were then filtered from the remainder. After filtering, gene counts were normalized using DESeq2 median of ratios method, and *log*_*2*_ *(x+1)* transformed. The 500 most variable genes in the count space were used for the decomposition in a tensor framework of genes‐by‐participant‐by‐day. Only participants with data for all days in the study were used. The final dimensions of the tensor were 500 × 54 × 4. The decomposition was performed using CP‐OPT with a non‐negative lower bound in the Tensor Toolbox package (Tensor Toolbox for MATLAB, 2021) in MatlabR2020a, which is a gradient‐based optimization method that has been shown to be more accurate than CP‐ALS (alternating least squares) and more efficient than CP‐NLS (nonlinear alternating least squares) (Acar et al., 2011). In order to determine the optimal rank for follow‐on analysis, the decomposition was repeated with random initializations, and the normalized Frobenius error and Similarity score were computed for each decomposition (see Appendix S1 for additional details). A rank 5 model was chosen as it represented the highest Similarity score before a drop with increasing rank and decreased component integrity. Top scoring genes were considered as the top 5% scoring genes for each component based on assessment of a scree plot of gene‐by‐component scores for each component (Figure S5). All code related to the transcriptional analysis can be found in *https://bitbucket.org/kleinstein/projects/src/master/Konstorum2022/*.

### 4.9 Analysis and modeling of flow cytometry markers along with comparison to RNASeq outputs

Longitudinal analysis of flow cytometry and RNA seq marker activation levels were conducted using generalized linear mixed effect models (PROC GLIMMIX) using a lognormal distribution and identity link function. Activation levels for each marker were modeled as a function of group, day of observation, and a group by day interaction adjusted for biological sex, the use of NSAID medication, and daily aspirin use (which corresponds to the same participants that were on daily aspirin and/or prescription medications as identified by the dAspirinplus category). A spatial exponential covariance structure was included to account for within‐participant correlations across repeated measurements at unequal days between observations. Marginal estimates were computed using LSMEANS. These analyses were generated using SAS/STAT software, Version 15.2. Copyright © 2020 SAS Institute Inc. SAS and all other SAS Institute Inc. product or service names are registered trademarks or trademarks of SAS Institute Inc., Cary, NC, USA.

## AUTHOR CONTRIBUTIONS

A.C.S., S.H.K., and R.R.M. were involved in conceptualization and supervision of the study, with T.M.G. providing expertise on frailty assessment and studies on frail older adults. M.T.R. provided essential help with platelet biological experiments. A.N. and S.M. recruited participants and collected clinical data and samples. S.M. was responsible for sample preparation, RNA preparation and flow cytometric analyses of platelet function. A.C.S., S.H.K., R.R.M. Y.Z., A.K., B.V.W., and H.G.A. contributed to methodology for analysis and interpretation of results. A.M. performed alignment of the RNAseq data and S.T. and A.M. were responsible for data curation. T.P.B., R.B.B., and D.G.C. performed and analyzed the HAI assays. A.K., B.V.W. and H.G.A. performed a formal analysis of the data. A.K., S.H.K., and A.C.S. drafted the initial manuscript. All authors contributed to revision and editing of the manuscript.

## CONFLICTS OF INTEREST

SHK receives consulting fees from Peraton.

## Supporting information


Figure S1–S5

Table S1–S12

Appendix S1

Extended Data Figures S1‐S4

## Data Availability

Flow cytometry and RNA‐seq data are available via ImmPort (https://www.immport.org) under study accession number SDY1393.
