# Supplementary material for: Platelet response to influenza vaccination reflects effects of aging
Source: Aging Cell. 2023 Jan 19;22(2):e13749. doi: 10.1111/acel.13749 (PMC9924941; doi:10.1111/acel.13749)
Supplement: Supplementary file 1 — Figure S1–S5 [file ACEL-22-e13749-s004.pdf]

Supplementary Figures

Platelet response to influenza vaccination reflects effects of aging

Konstorum A., Mohanty S., Zhao Y., Melillo A., Vander Wyk B., Nelson A., Tsang S., Blevins T.P., Belshe R.B., Rondina M.T., Gill T.M., Montgomery R.R., Allore H.G., Kleinstein, S.H., Shaw A.C.

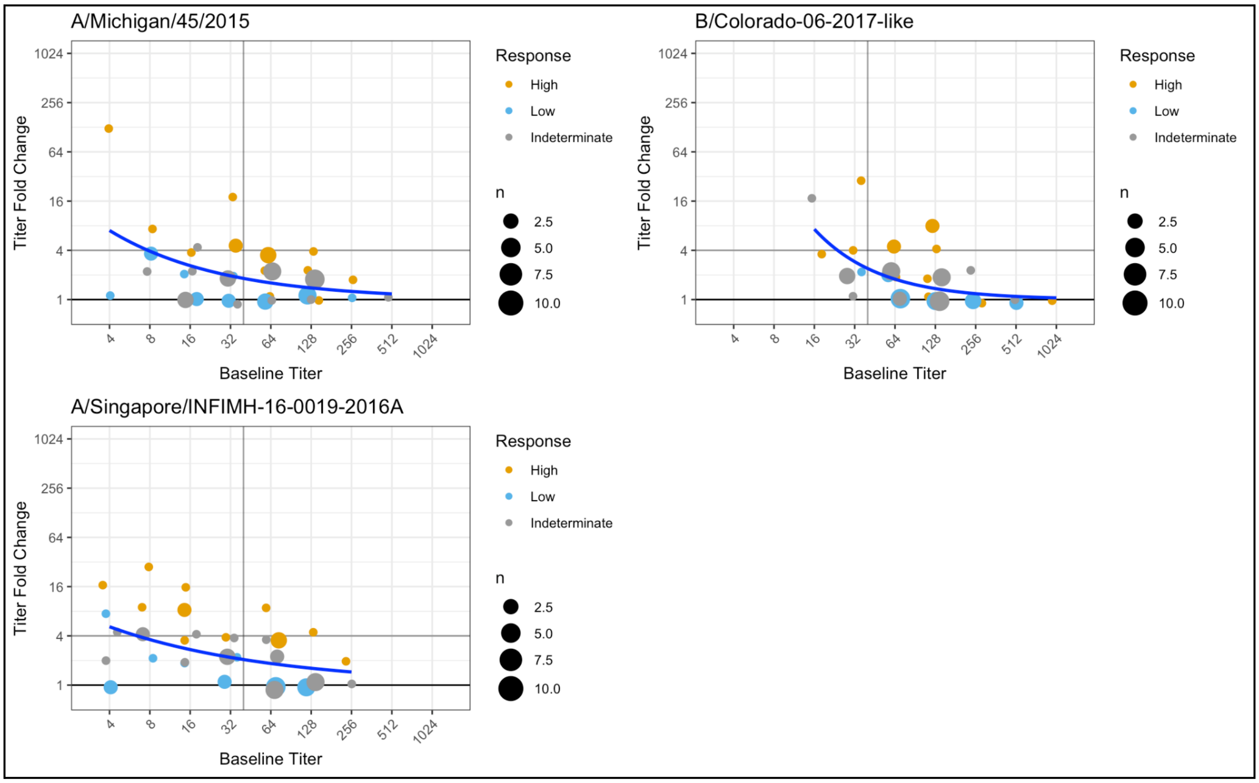

Supplementary Fig. 1: maxRBA calculation for HAI titers. An exponential curve (blue) is fit to the data, and the residual is used to stratify subjects. maxRBA is calculated using the maximum residual across all vaccine strains as described in the Methods.

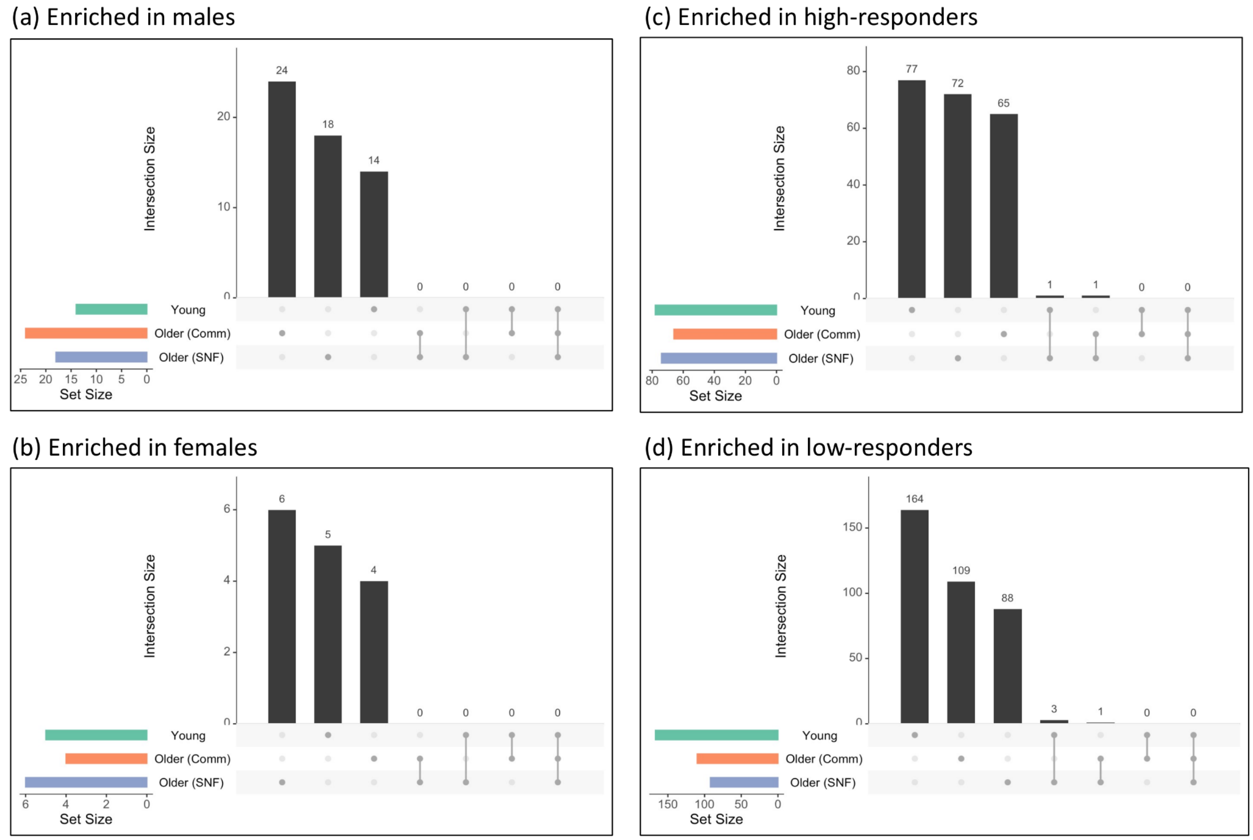

Supplementary Fig. 2: Unique and overlapping differentially expressed RNAs at baseline for each of Young, Older (Comm), and Older (SNF) adults with respect to (a) male v. female, enriched in males, (b) male v. female, enriched in females, (c) high- v. low-responders, enriched in high-responders and (d) high- v. low-responders, enriched in low-responders. For (c,d) high- and low-responders counts per group are Young (high, n=7; low, n=9), Older (Comm) (high, n=5; low, n=8), Older (SNF) (high, n=5; low, n=4).

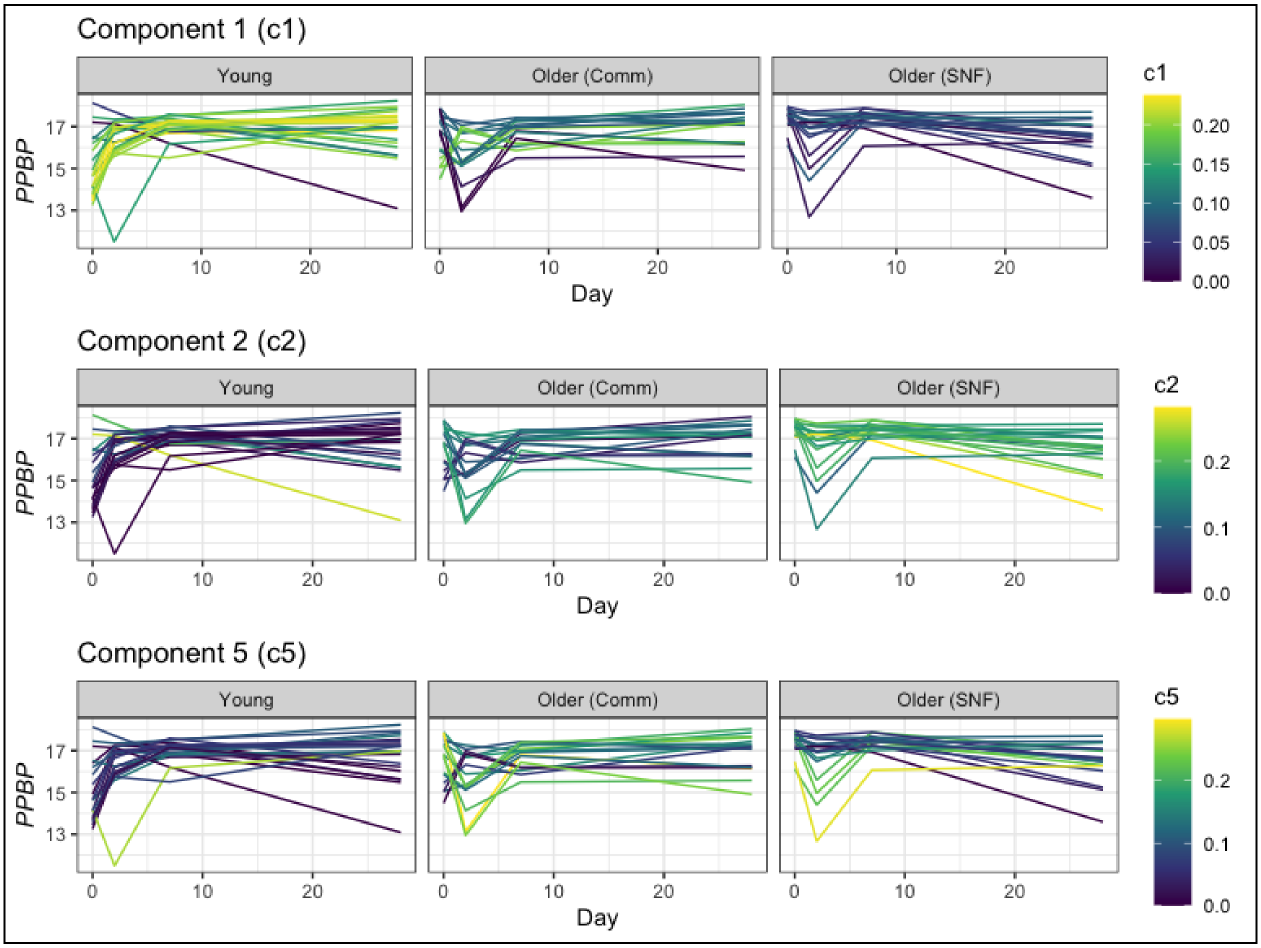

Supplementary Fig. 3: Trajectory of *PPBP* colored by component 1, 2, and 5 scores, respectively, for the three different groups.

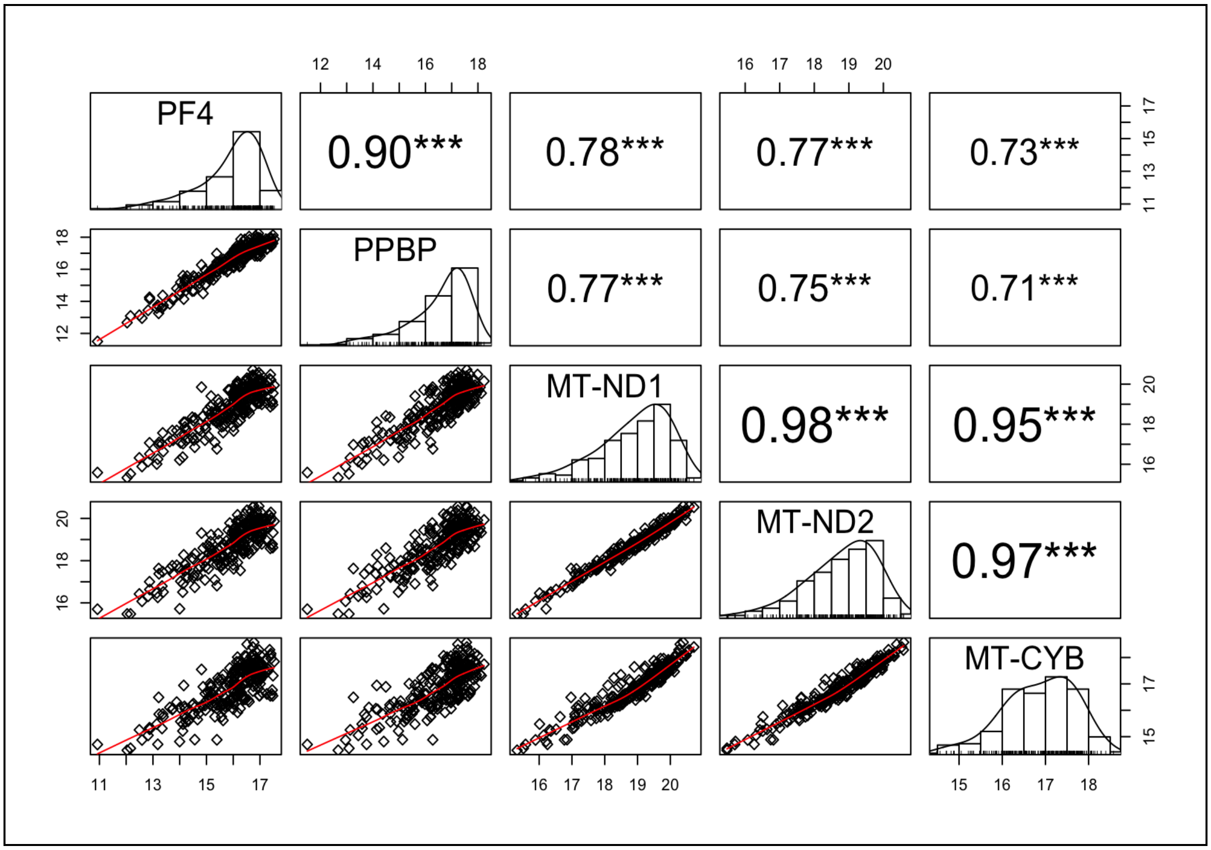

Supplementary Fig. 4: Mitochondrial genes, represented here by *MT-ND1*, *MT-ND2*, and *MT-CYB* and platelet activation RNAs, represented here by *PF4* and *PPBP* are significantly correlated across all samples, baseline and post-vaccination.

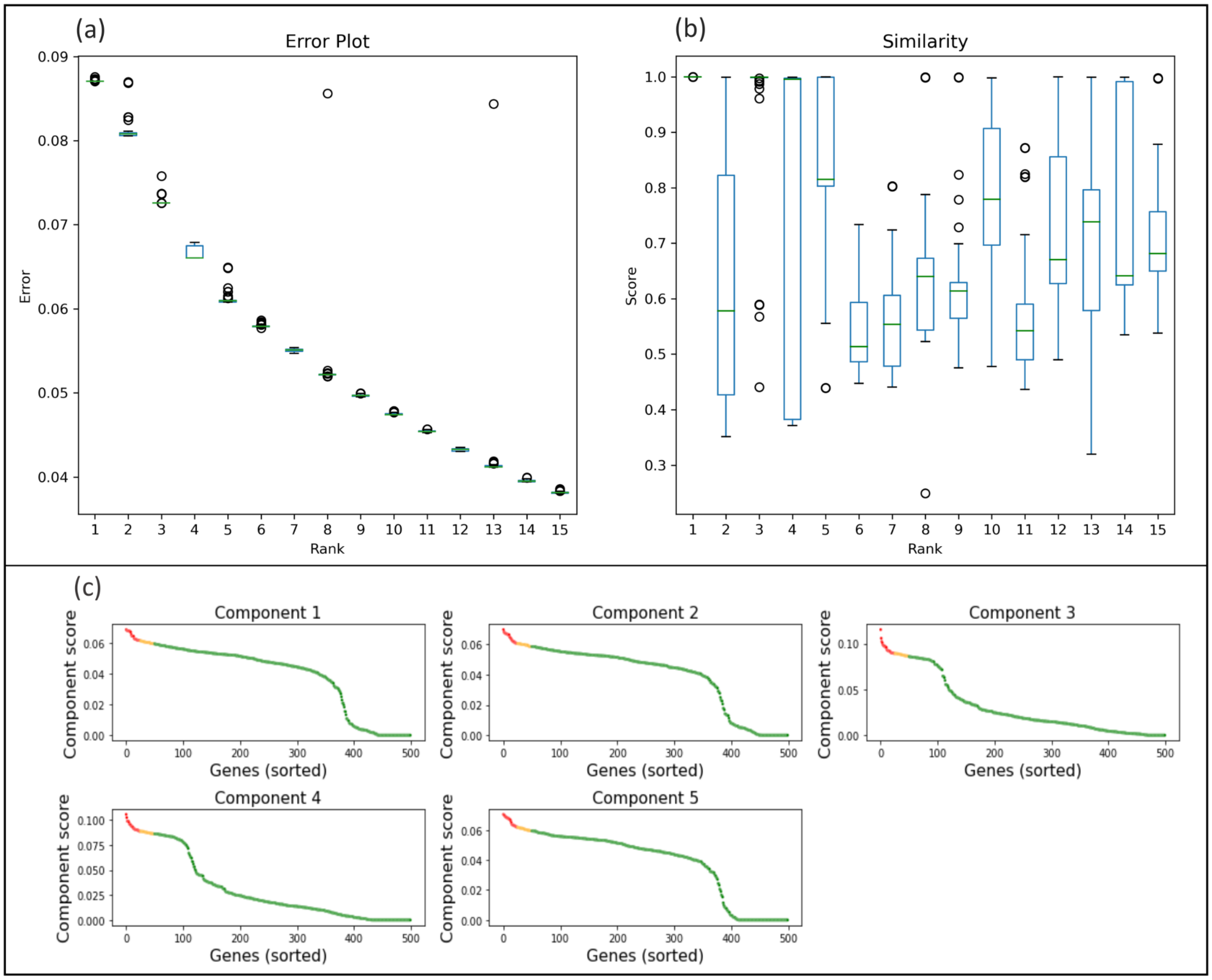

Supplementary Fig. 5: Determining optimal rank and model for a time-course platelet vaccination non-negative CP tensor decomposition. (a) Normalized Frobenius error and the (b) Similarity score were calculated for decompositions of ranks *R*=1-15, over 100 random initial conditions for each rank. (c) Gene RNAs ranked by component score for each component of a rank-5 decomposition. Top 5% of scores are in red, top 10% scores are in red and yellow, and remaining scores in green.
