# Supplementary material for: Platelet response to influenza vaccination reflects effects of aging
Source: Aging Cell. 2023 Jan 19;22(2):e13749. doi: 10.1111/acel.13749 (PMC9924941; doi:10.1111/acel.13749)
Supplement: Supplementary file 4 — Extended Data Figures S1‐S4 [file ACEL-22-e13749-s001.pdf]

Extended Data Figures

Platelet response to influenza vaccination reflects effects of aging

Konstorum A., Mohanty S., Zhao Y., Melillo A., Vander Wyk B., Nelson A., Tsang S., Blevins T.P., Belshe R.B., Rondina M.T., Gill T.M., Montgomery R.R., Allore H.G., Kleinstein, S.H., Shaw A.C.

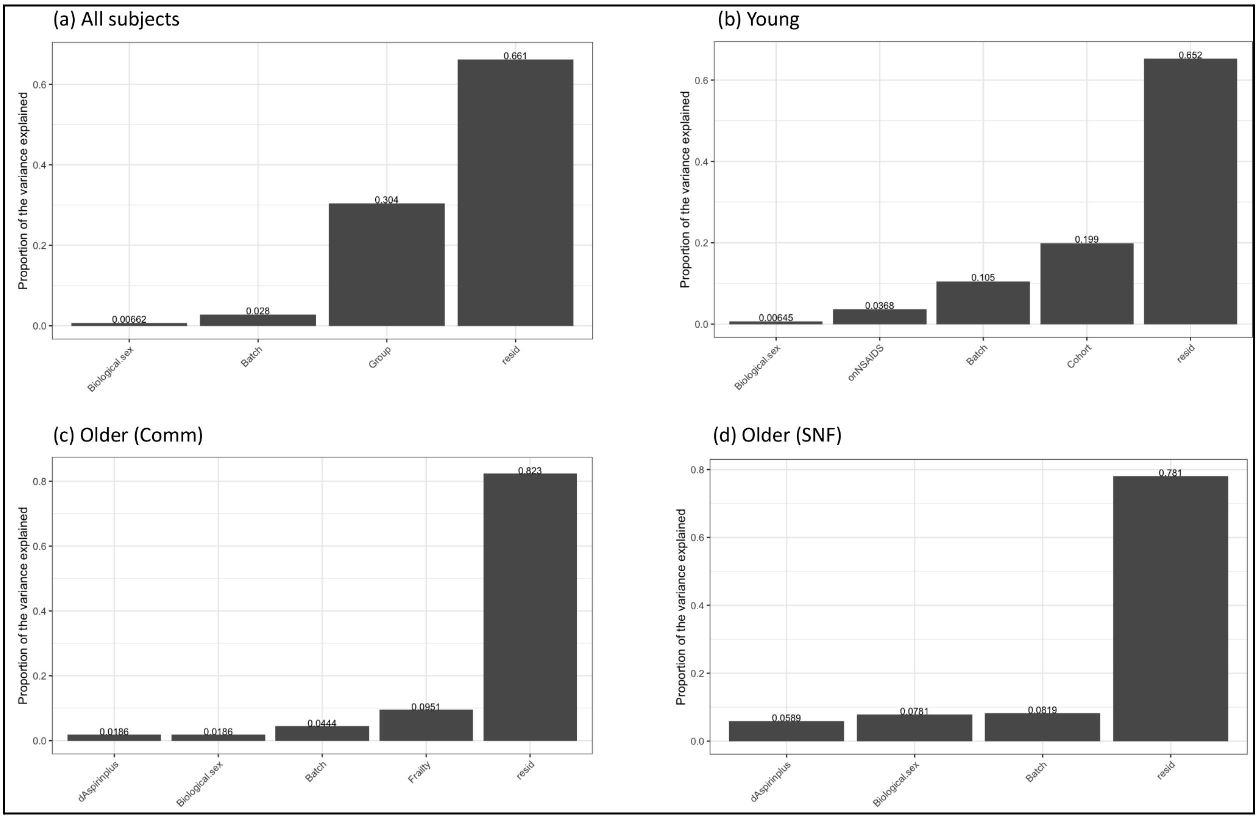

Extended Data Fig. 1: Principal Variance Component Analysis (PVCA) for covariates associated with (a) all participants and the (b) Young, (c) Older (Comm) and (d) Older (SNF) groups. Biological.sex: biological sex of participant; Batch: sequencing run; Group: Young, Older (Comm), or Older (SNF) group assignment; onNSAIDs: whether participant was on NSAIDs; dAspirinplus: whether participant was on daily aspirin or prescription anti-platelet medication; Cohort: site location for Young adult sample collection; Frailty: frailty classification (non- or pre-frail) for Older (Comm) adults; resid: undefined residual effects.

(a) Correlation of platelet activation and MT genes

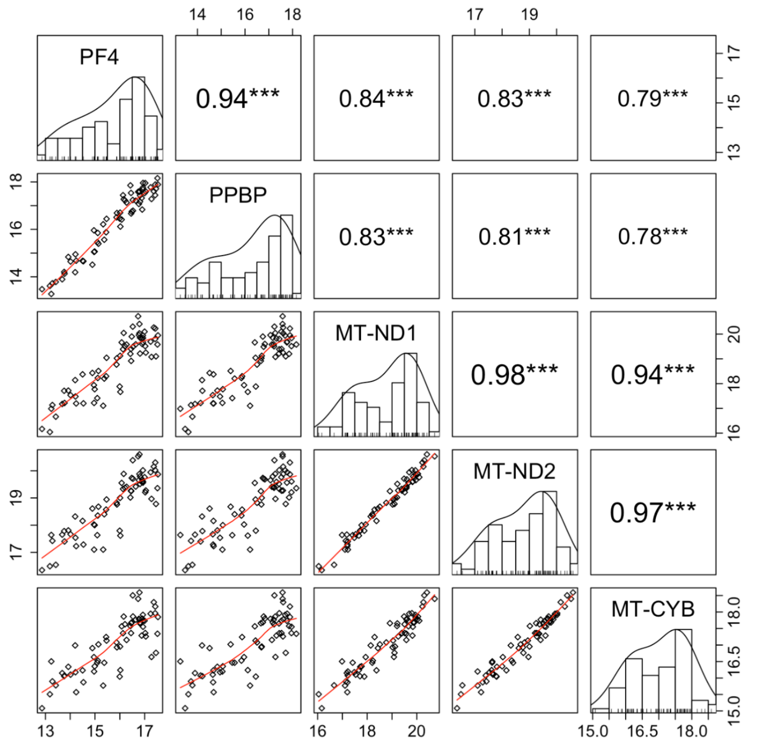

(b) Older (Comm) v. Young

Enriched in Older (Comm)

Pathway name	adj. p value
Respiratory electron transport	9.80E-04
Respiratory electron transport, ATP synthesis by chemiosmotic coupling, and heat production by uncoupling proteins	1.08E-03
The citric acid (TCA) cycle and respiratory electron transport	1.00E-02
Complex I biogenesis	3.67E-02

Extended Data Fig. 2: (a) Mitochondrial genes are correlated with expression of platelet activation RNAs. (b) Pathways that involve mitochondrial genes are enriched in Older (Comm) v. Young.

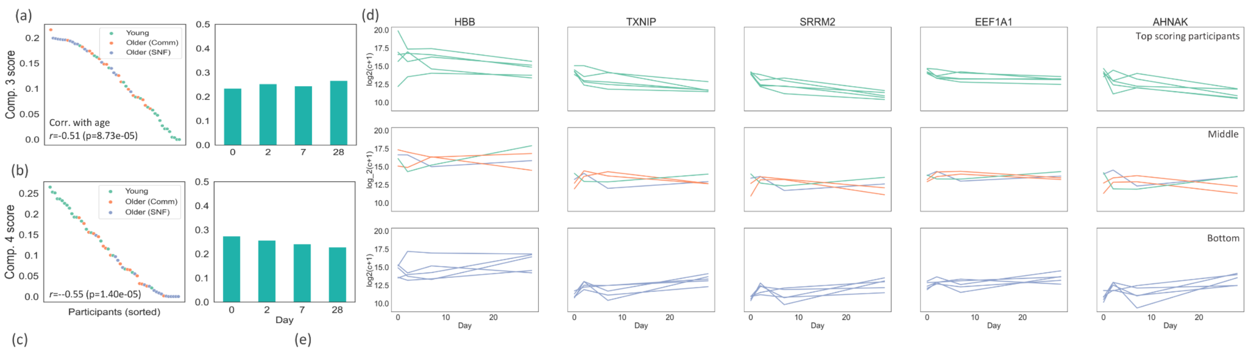

(c)

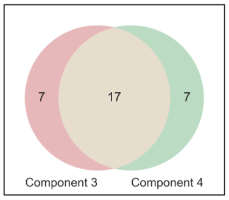

(e)

Pathway	*adj. p value*
Peptide chain elongation	1.44E-05
Eukaryotic Translation Elongation	1.80E-05
Translation	4.94e-03
Viral mRNA Translation	4.79e-02

Extended Data Fig. 3: Tensor components relate to translation pathways and age group. (a,b) Sample and Day scores for Components 3 and 4, respectively, (c) Venn diagram of overlapping RNAs in Components 3 and 4, (d) Expression levels for the top, middle, and bottom 5 scoring subjects and top 5 scoring RNAs in component 4, (e) Overrepresented Reactome pathways shared by the two Components (adj. p value < 0.05).

(a)

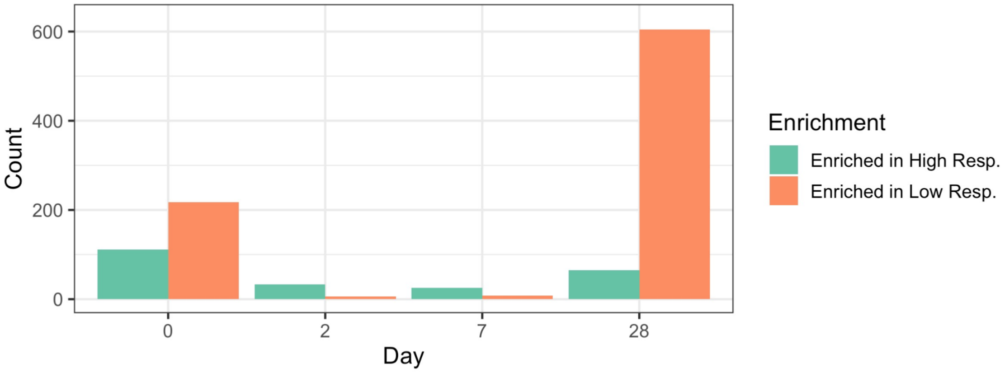

(b)

**Pathway name**	**adj. p value**
RHO GTPase Effectors	4.49E-12
Signaling by Rho GTPases, Miro GTPases and RHOBTB3	6.71E-11
Signaling by Rho GTPases	6.71E-11
RHO GTPases activate PKNs	6.71E-11
Platelet activation, signaling and aggregation	3.84E-09
RUNX1 regulates genes involved in megakaryocyte differentiation and platelet function	6.30E-09
Hemostasis	1.43E-08
Transcriptional regulation of granulopoiesis	3.97E-07
Transcriptional regulation by RUNX1	1.27E-06
Amyloid fiber formation	1.40E-06

Extended Data Fig. 4: (a) Total number of differentially expressed RNAs (adj. p value < 0.10) between Young high- vs. low-responders for all protein-coding RNAs, (d) Top ten overrepresented pathways of RNAs enriched in low responders at day 28.
